# Exogenous Glutamine in Respiratory Diseases: Myth or Reality?

**DOI:** 10.3390/nu8020076

**Published:** 2016-02-04

**Authors:** Gisele P. Oliveira, Marcelo Gama de Abreu, Paolo Pelosi, Patricia R. M. Rocco

**Affiliations:** 1 Laboratory of Pulmonary Investigation, Carlos Chagas Filho Institute of Biophysics, Federal University of Rio de Janeiro, Centro de Ciências da Saúde, Av. Carlos Chagas Filho, 373, Bloco G-014, Ilha do Fundão, Rio de Janeiro, RJ 21941-902, Brazil; giselepoliv@gmail.com; 2 Department of Anesthesiology and Intensive Care Therapy, Pulmonary Engineering Group, University Hospital Carl Gustav Carus, Technische Universität, Fetscherstrasse 74, 01307 Dresden, Germany; mgabreu@uniklinikum-dresden.de; 3 Department of Surgical Sciences and Integrated Diagnostics, Istituto di Ricovero e Cura a Carattere Scientifico, Research Hospital (IRCCS AOU), San Martino Istituto Nazionale Tumori, National Cancer Institute (IST), University of Genoa, 16132 Genoa, Italy; ppelosi@hotmail.com

**Keywords:** glutamine, cystic fibrosis, asthma, chronic obstructive pulmonary disease, acute respiratory distress syndrome, lung cancer

## Abstract

Several respiratory diseases feature increased inflammatory response and catabolic activity, which are associated with glutamine depletion; thus, the benefits of exogenous glutamine administration have been evaluated in clinical trials and models of different respiratory diseases. Recent reviews and meta-analyses have focused on the effects and mechanisms of action of glutamine in a general population of critical care patients or in different models of injury. However, little information is available about the role of glutamine in respiratory diseases. The aim of the present review is to discuss the evidence of glutamine depletion in cystic fibrosis (CF), asthma, chronic obstructive pulmonary disease (COPD), acute respiratory distress syndrome (ARDS), and lung cancer, as well as the results of exogenous glutamine administration in experimental and clinical studies. Exogenous glutamine administration might be beneficial in ARDS, asthma, and during lung cancer treatment, thus representing a potential therapeutic tool in these conditions. Further experimental and large randomized clinical trials focusing on the development and progression of respiratory diseases are necessary to elucidate the effects and possible therapeutic role of glutamine in this setting.

## 1. Introduction

Glutamine is the most abundant amino acid in humans, contributing to approximately 60% of the free amino acid pool in muscle and approximately 20% of the amino acid pool in plasma [1]. Glutamine is a nutrient that participates in various cellular processes, including energy and nucleotide formation [2], redox homeostasis [3], acid-base balance [4], and glucose metabolism [5]. Since most tissues can synthesize glutamine, it is not considered an essential amino acid. However, it has been hypothesized that, during catabolic stress, glutamine may become conditionally essential, e.g., during intense exercise [6] and critical illness [7,8]. Glutamine requirements appear to exceed the mammalian body’s capacity to synthesize this amino acid, leading to a decrease of plasma and intracellular glutamine concentrations. This represents the rationale for administration of exogenous glutamine to critically ill patients. Several recent meta-analyses have evaluated the effects of parenteral and enteral glutamine in critical care settings. One meta-analysis investigated the potential benefits of parenteral glutamine supplementation in a general population of intensive care unit (ICU) patients, and found that, when combined with nutritional support, glutamine supplementation led to a significant reduction in hospital mortality and hospital length of stay [9]. A systematic review and meta-analysis of randomized clinical trials evaluating the effects of intravenous glutamine dipeptide supplementation in patients undergoing elective abdominal surgery observed a positive effect of glutamine on length of stay, without affecting the rate of complications [10]. Another systematic review studied the impact of enteral glutamine supplementation in patients with critical illness (defined as ICU admission). Compared to an isonitrogenous control, enteral glutamine had no impact on outcomes in this general population of ICU patients, but reduced in-hospital mortality in burn patients [11]. In a systematic review and meta-analysis including clinical trials of patients admitted to ICU that used both parenteral and enteral glutamine, supplementation was not found to reduce in-hospital mortality, ICU mortality, or the rate of infection [12]. Thus, the effect of glutamine appears to depend on the route of administration, and parenteral supplementation may lead to better results. However, the majority of meta-analyses conducted to date evaluated the use of glutamine in general populations of ICU patients.

As respiratory diseases are usually accompanied by a deregulated inflammatory response [13,14,15], oxidative stress [16], comorbidities such as malnutrition [17,18], and a catabolic state [18,19], they may feature glutamine depletion. However, to our knowledge, no review has specifically addressed the effects of glutamine therapy on respiratory diseases. The aim of the present review is to discuss the role of glutamine depletion in cystic fibrosis (CF), asthma, chronic obstructive pulmonary disease (COPD), acute respiratory distress syndrome (ARDS), and lung cancer, as well as the results of exogenous glutamine administration in these conditions.

## 2. Is Glutamine Depleted in Critical Illness and Respiratory Diseases?

Clinical interest in glutamine began in 1975, when intracellular free glutamine in skeletal muscle was found to be markedly reduced in the stress response to surgery, trauma, and inflammatory states [20]. Roth and colleagues [7] showed that muscle glutamine levels yielded the highest discriminant power for classification of non-survivors and survivors of prolonged abdominal sepsis on the second day after laparotomy. Non-survivors presented low concentrations of muscle glutamine and high levels of branched-chain amino acids, possibly indicating inhibited intracellular glutamine formation in muscle tissue. A later study performed in a cohort of seriously ill patients non-electively admitted to the ICU demonstrated higher hospital mortality in a group of patients with low plasma glutamine concentrations [8]. A low plasma glutamine concentration at ICU admission was also considered an independent risk factor for post-ICU mortality [21]. Such evidence led to the hypothesis that exogenous glutamine could supply this amino acid for use by cells that metabolize it, thus activating tissue protection pathways and improving outcomes in severely ill patients. On the other hand, a multicenter randomized controlled trial of 66 critically ill patients with multi-organ failure did not find evidence of glutamine deficiency [22]. Plasma glutamine levels at discharge were also within normal limits and were not predictive of mortality [23]. Additionally, a high glutamine concentration at ICU admission may also indicate a negative outcome [21].

Some studies have investigated glutamine status specifically in the setting of respiratory diseases. The first study to investigate amino acid profile in COPD revealed reduced values of plasma arterial glutamine in emphysema patients with evidence of muscle wasting [24]. Decreased levels of skeletal muscle glutamine, as well as decreased muscle-to-arterial glutamine gradients, were also found in patients with emphysema [25]. Unexpectedly, Pouw and colleagues [26] found low plasma glutamine levels, but increased muscle glutamine concentration. Similarly, a high level of muscle glutamine was found in patients with COPD as compared with control individuals [27]. Such conflicting results could express striking differences in intracellular amino acid profile between the chronic bronchiolitis and emphysema phenotypes of COPD.

In CF patients, a marked decrease of glutamine content in circulating neutrophils was observed, with normal plasma values [28]. Moreover, CF children with severe mutations in cystic fibrosis transmembrane conductance regulator (CFTR) had even lower neutrophil glutamine content compared to children with mild mutations [28]. The presence of normal glutamine plasma values in CF patients suggests that a systemic glutamine deficiency does not occur in these patients, indicating that the presence of a chronic inflammatory response *per se* does not lead to neutrophil glutamine depletion. Moreover, the CFTR mutation could be directly correlated with neutrophil glutamine depletion. Glutamine is involved in several key metabolic processes in neutrophils, which are essential to sustain a number of different cellular processes, including motility, respiratory burst, and secretion of cytoplasmic proteolytic enzymes and immunomodulatory compounds involved in the initiation of phagocytosis and bacterial killing [29].

In adult patients with asthma who received at least one dose of an inhaled corticosteroid daily, plasma glutamine levels were not decreased as compared to those of controls. However, a trend toward reduced odds of asthma diagnosis was noted for subjects with higher concentrations of glutamine [30]. Such observations were supported by another investigation, which found significantly lower plasma concentrations of glutamine in mild asthmatics than in healthy controls [31]. Conversely, asthma patients exhibited increased serum levels of glutamine, which is involved in hypermethylation, response to hypoxia, and immune reaction [32].

In cancer, glutamine requirements are particularly significant in malignant cells, inducing a net glutamine flux from host to tumor. In patients with progressive cancer, the liver releases glutamine to provide additional circulating glutamine for tumor cells [33]. The dependency of lung cancer cells on glutamine for growth, survival, and cell-cycle progression is well documented [34]. In the majority of patients with cancer, glutamine depletion develops with time, both from the disease process itself and from the catabolic effects of antineoplastic therapies [35,36].

To our knowledge, no studies have investigated plasma glutamine levels specifically in ARDS patients.

In short, reductions of plasma glutamine concentration may fluctuate in critical illness, as well as in some respiratory diseases. The altered amino acid flux that occurs in metabolic stress may be reflected by changes in plasma amino acid concentration and, consequently, by systemic glutamine depletion.

## 3. The Role of the Lungs in the Glutamine Pool

Organ-specific glutamine metabolism has been studied in humans and in animal models of physiological stress by measuring the arteriovenous difference in plasma glutamine content. In healthy subjects, the plasma glutamine pool results from the release of this amino acid by skeletal muscles [1]. In rats, the lungs and muscle tissue are comparable in terms of glutamine production [37]. In humans, lungs also have a capacity for marked glutamine release, mainly during stress [38,39], thus contributing to maintain glutamine homeostasis. Unlike skeletal muscle, which contains substantial quantities of free glutamine, the lung primarily contributes to *de novo* synthesis of glutamine from glutamate and ammonia, a reaction catalyzed by the enzyme glutamine synthetase [40,41]. Stress-induced release of glutamine from the lung is a consequence of glucocorticoid signaling and other mechanisms [40,41]. Although the resulting arteriovenous difference is relatively small, the overall glutamine release is significant because of massive pulmonary perfusion. Moreover, alterations in lung glutamine metabolism are characteristic of critical illness [38].

One study found that lung glutamine production was 850% higher in septic surgical patients compared with preoperative controls [38]. A later investigation added further support to the hypothesis that the lungs play an important role in glutamine homeostasis during sepsis [39]. However, pulmonary glutamine release was less than that reported in the previous publication [38], which could be explained by differences in patient population. Furthermore, this study showed diminished pulmonary glutamine efflux in the presence of lung infiltrates, suggesting an increase in local use rather than a decrease in glutamine synthesis capacity.

## 4. Glutamine Therapy in Respiratory Diseases

### 4.1. Glutamine in the Acute Respiratory Distress Syndrome

ARDS is a heterogeneous syndrome that encompasses lung injury in the setting of underlying illnesses that may cause either direct or indirect damage to the lung. It is characterized by deregulated inflammation, inappropriate accumulation and activity of leukocytes and platelets, uncontrolled activation of coagulation pathways, and altered permeability of alveolar endothelial and epithelial barriers [13]. The pathophysiology of ARDS differs by type of primary insult, resulting in distinct molecular phenotypes, and, consequently, different response to therapies [42,43]; for example, ARDS secondary to direct lung injury is characterized by a molecular phenotype consistent with more severe lung epithelial injury and less severe endothelial injury than in ARDS caused by an indirect insult [44].

Although considerable progress has been made in understanding the pathogenesis and pathophysiology of ARDS, it is a complex syndrome with a broad clinical phenotype, and no pharmacological therapies effective in reducing mortality are available. In this context, most experimental studies have investigated the effects and mechanisms of action of glutamine in many different models of ARDS, while no clinical trial has focused on the development of ARDS in critically ill patients. Moreover, although bacterial or viral pneumonia (producing a direct insult to the lung) is the most common cause of ARDS [45], the majority of experimental studies investigating the impact of glutamine in ARDS used models of indirect lung injury.

**Figure 1:**
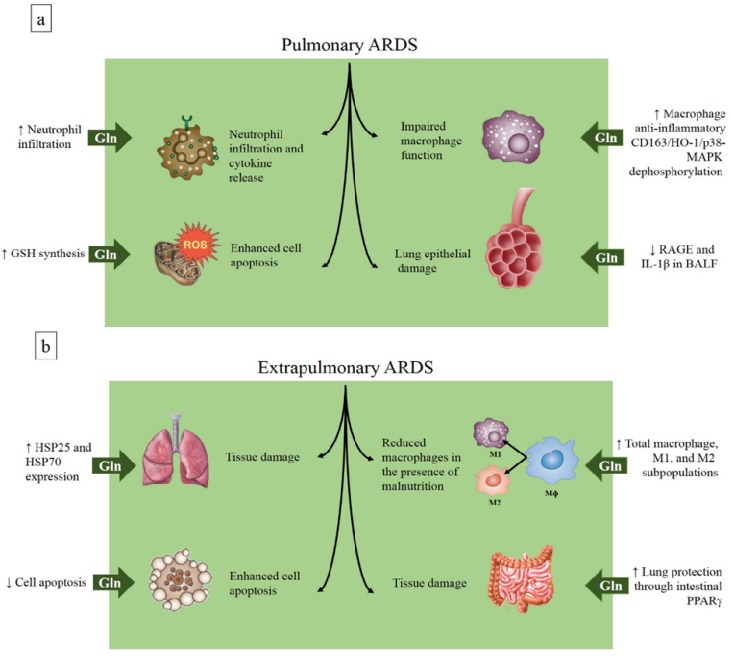
Effects of glutamine in models of pulmonary (**a**) and extrapulmonary (**b**) acute respiratory distress syndrome. ARDS, acute respiratory distress syndrome; BALF, bronchoalveolar lavage fluid; CD163/HO-1/p38-MAPK, CD163/heme-oxygenase-1/p38-mitogen-activated protein kinase; Gln, glutamine; GSH, reduced glutathione; HSP, heat shock protein; IL, interleukin; PPAR, peroxisome proliferator-activated receptor; ROS, reactive oxygen species; M, macrophage; RAGE, receptor for advanced glycation end-products; ROS, reactive oxygen species.

In a model of extrapulmonary ARDS induced by cecal ligation and puncture (CLP), early intravenous glutamine administration significantly reduced mortality and attenuated lung injury [46]. These benefits were associated with enhanced heat shock protein (HSP)-70 and HSP-25 expression in lung tissue. Similarly, a study by our group found that early intravenous glutamine administration reduced lung injury in a model of CLP-induced sepsis; in addition, lessened diaphragmatic damage and distal organ apoptosis were observed [47]. Interestingly, when this model of CLP-induced sepsis was combined with malnourishment, the benefits of glutamine administration were maintained, and may thus be associated with activation of macrophages in the lung, since malnourishment could compromise the immune response to infection [48]. In a model of hind limb ischemia/reperfusion (IR) injury, a single intravenous dose of glutamine administered immediately after limb ischemia reduced the local and systemic inflammatory response and attenuated lung injury [49]. Besides the intravenous route, enteral administration of glutamine provided significant protection against gut injury and inflammation, with similar lung protection when administered at the onset of reperfusion, in a model of IR-induced intestinal damage. In mice lacking intestinal epithelial cell-specific peroxisome proliferator-activated receptor (PPAR)-γ, glutamine lost its protective effects, suggesting a critical role of PPARγ in the protection provided by enterally administered glutamine [50] (Figure 1b). In fact, a similar dose of glutamine or alanyl-glutamine dipeptide administered through the enteral *versus* the parenteral route has less effect on plasma glutamine levels, possibly owing to splanchnic extraction [51]. Thus, since glutamine administration was performed immediately at the onset of reperfusion, it is likely that the protective effect of the treatment on the lung was induced by decreased intestinal damage. Unfortunately, in the cited study [50], the authors did not report plasma glutamine levels and the direct effect on lung cells could not be excluded.

On the other hand, the results from pulmonary models of ARDS are controversial. In mice pretreated for 10 days with a glutamine-supplemented diet and subjected to ARDS induced by intratracheal instillation of *Escherichia coli* lipopolysaccharide (*E. coli* LPS), higher inflammatory cytokine production and increased neutrophil recruitment at the early stage of ARDS were observed as compared to controls [52]. In a similar model of LPS-induced (pulmonary) ARDS, rats received intravenous glutamine as alanyl-glutamine dipeptide (0.75 g/kg body weight) at the same time of LPS administration. Two and 18 h after induction of lung injury, glutamine prevented morphological changes in the lung parenchyma and neutrophil infiltration in the alveolar space, enhanced reduced glutathione (GSH) synthesis, attenuated interleukin (IL)-8 release in lung tissue, and prevented higher CD11b expression in blood neutrophils [53]. Another study performed in mice fed a glutamine-supplemented diet (0.8 g/kg body weight from feed conversion) 10 days before intratracheal administration of hydrochloric acid plus *E. coli* LPS-induced ARDS showed that glutamine pretreatment reduced levels of the receptor for advanced glycation end-products (RAGE) and interleukin (IL)-1β in bronchoalveolar lavage fluid (BALF), with corresponding decreases in mRNA expression [54]. In a two-hit model of hydrochloric acid aspiration plus injurious mechanical ventilation, intravenous glutamine, administered 30 min before the randomization for recruitment maneuver and soon after inducing acid aspiration, improved lung morphofunction as well as neutrophil recruitment and cytokine production in lung tissue [55]. A 2015 study showed that rats pretreated with two doses of l-alanyl-l-glutamine (0.75 g/kg glutamine) by oral gavage before intratracheal instillation of IL-1 plus LPS had decreased lung damage, as reflected by decreases in BALF protein and lactate dehydrogenase. In addition, pretreatment increased the number of alveolar macrophages and activated the alveolar macrophage anti-inflammatory CD163/heme-oxygenase-1/p38-mitogen-activated protein kinase (CD163/HO-1/p38-MAPK) dephosphorylation pathways, although it did not decrease neutrophil recruitment into the lung [56] (Figure 1b, Table 1 and Table 2).

It is likely that different routes and modalities of glutamine administration (pretreatment *versus* immediately after the insult), as well as the time of the treatment may influence the results in experimental ARDS.

**Table 1:** Glutamine administration in ARDS models.

Reference	Animal Model	Form Administered	Time, Route of Administration	Dose	Main Effects
**Extrapulmonary**					
Singleton *et al.* 2005 [46]	CLP (Sprague-Dawley rats)	Alanyl-glutamine	1 h after injury, i.v.	0.75 g·kg^−1^	Reduced mortality, attenuated occurrence of lung injury
Oliveira *et al.* 2009 [47]	CLP (Wistar rats)	Alanyl-glutamine	1 h after injury, i.v.	0.75 g·kg^−1^	Attenuated lung, diaphragm, and distal organ injury
Oliveira *et al.* 2014 [48]	CLP + malnourishment (Wistar rats)	Alanyl-glutamine	1 h after injury, i.v.	0.75 g·kg^−1^	Attenuated lung and distal organ injury
Shih *et al.* 2015 [49]	Limb IR (C57BL/6 mice)	Alanyl-glutamine	Immediately after the injury, i.v.	0.75 g·kg^−1^	Reduced systemic inflammation, minimized lung injury
Peng *et al.* 2015 [50]	Gut IR (C57BL/6J mice)	Glutamine	1 h after the ischemia, enteral	60 mM	Improved survival and protected lung against injury and inflammation
**Pulmonary**					
Hou *et al.* 2009 [52]	LPS i.t. (C57BL/6 mice)	Glutamine	Pretreatment, oral (supplemented diet)	25% total nitrogen	Increased lung inflammation
Zhang *et al.* 2009 [53]	LPS i.t. (Sprague-Dawley rats)	Alanyl-glutamine	Immediately after the injury, i.v.	0.75 g·kg^−1^	Protected alveolar barrier and attenuated inflammatory injury
Chuang *et al.* 2014 [54]	Hydrochloric acid + LPS i.t. (BALB/c mice)	Glutamine	Pretreatment, oral (supplemented diet)	0.8 g·kg^−1^	Inhibited RAGE expression and minimized lung injury
Lai *et al.* 2014 [55]	Hydrochloric acid + injurious mechanical ventilation (MV) (Sprague-Dawley rats)	Alanyl-glutamine	Immediately after the injury induced by hydrochloric acid and 30 min before MV, i.v.	0.75 g·kg^−1^	Improved oxygenation and lung mechanics, decreased tissue damage and inflammation
Fernandez-Bustamante *et al.* 2015 [56]	IL-1 + LPS i.t. (Sprague-Dawley rats)	Alanyl-glutamine	Pretreatment, oral gavage	0.75 g·kg^−1^	Decreased lung capillary damage

ARDS, acute respiratory distress syndrome; CLP, cecal ligation and puncture surgery; IL-1, interleukin-1; IR, ischemia/reperfusion; i.t., intratracheal; i.v., intravenous; LPS, lipopolysaccharide; RAGE, receptor for advanced glycation end-products.

**Table 2:** Regulatory mechanisms of glutamine in respiratory diseases.

**ARDS**
Enhances HSP-70 and HSP-25 expression
Inhibits apoptosis
Improves macrophage function
Reduces pro-inflammatory cytokine release
Decreases neutrophil infiltration
Enhances GSH synthesis
Reduces RAGE expression
Activates CD163/heme-oxygenase-1/p-38 MAPK dephosphorylation
**Asthma**
Suppresses cPLA2 activity
Reduces activation of p38 MAPK
**COPD**
Modulates IL-6, IL-8, and TNF-α release
Increases citrulline and arginine production
**CF**
No significant regulatory actions
**Cancer**
Improves immune function
Preserves GSH levels

ARDS, acute respiratory distress syndrome; CF, cystic fibrosis; COPD, chronic obstructive pulmonary disease; cPLA2, cytosolic phospholipase A2; GSH, reduced glutathione; HSP, heat shock protein; IL, interleukin; p38 MAPK, p38 mitogen-activated protein kinase; RAGE, receptor for advanced glycation end-products; TNF, tumor necrosis factor.

### 4.2. Glutamine in Asthma

Asthma is a heterogeneous disease, usually characterized by chronic airway inflammation. It has been defined as a history of respiratory symptoms—such as wheezing, shortness of breath, chest tightness, and cough—that vary over time and in intensity, together with variable expiratory airflow limitation. In both adults and children, asthma is the most common chronic respiratory disease, affecting 1% to 18% of the population in different countries [57]. An important molecular mechanism of asthma is type 2 inflammation, which occurs in most (but not all) patients with this condition. Airway type 2 immune responses are mainly mediated by eosinophils, mast cells, basophils, T helper (Th) 2 cells, group 2 innate lymphoid cells (ILC2s), and IgE-producing B cells [14].

**Figure 2:**
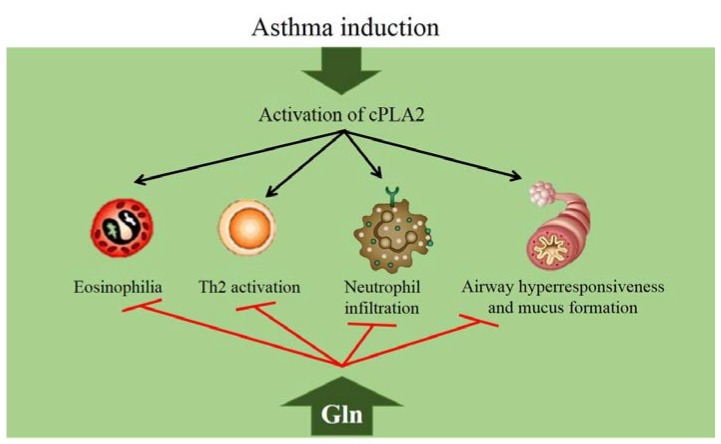
Effects of glutamine in asthma models. cPLA2, cytosolic phospholipase A2; Gln, glutamine; Th, T helper cell.

In a murine model of asthma, intraperitoneal administration of L-glutamine suppressed airway eosinophilia, mucus formation, and airway type 2 cytokine production, as well as late airway hyperresponsiveness. This effect was explained to be at least partly caused by the ability of glutamine to suppress the activity of cytosolic phospholipase A2 (cPLA2) [58]. Phospholipase A2 (PLA2) enzymes catalyze hydrolysis of membrane phospholipids to produce arachidonic acid, which is subsequently metabolized into potent lipid mediators of inflammation, mainly through the cyclo-oxygenase and lipoxygenase pathways, to produce prostaglandins, thromboxanes, and leukotrienes [59]. These mediators, in turn, have been shown to induce airway eosinophilia and bronchoconstriction. Indeed, control of PLA2 activity has been proposed as a mechanism for treating inflammatory/allergic respiratory diseases [60]. A later study investigated the effects and mechanisms of glutamine in suppressing neutrophil recruitment into the airways in a murine model of asthma. Glutamine suppressed airway neutrophilia by inhibiting cPLA2 phosphorylation, at least in part through deactivation of p38 MAPK in the asthmatic lungs [61] (Figure 2). In severe asthma, neutrophils are believed to play an important role in disease maintenance and during exacerbations, and these patients are steroid-resistant [62]. The precise mechanism of how glutamine inhibits cPLA2 phosphorylation was further analyzed and it was determined that glutamine dephosphorylates cPLA2 indirectly, through MAPK phosphatase-1 (MKP-1)-mediated p38 dephosphorylation [63].

### 4.3. Glutamine and COPD

Chronic obstructive pulmonary disease is a preventable and treatable disease characterized by persistent airflow limitation, which is usually progressive and associated with an enhanced chronic inflammatory response to noxious particles or gases in the airways and lungs. The chronic airflow limitation characteristic of COPD is caused by a mixture of small-airway disease (obstructive bronchiolitis) and parenchymal destruction (emphysema), with the relative contribution of each varying from person to person. Chronic inflammation causes structural changes and narrowing of the small airways, whereas destruction of the lung parenchyma, also by inflammatory processes, leads to the loss of alveolar attachments to the small airways and decreases lung elastic recoil; these changes, in turn, limit the ability of the airways to remain open during expiration [64]. In many patients, COPD is associated with several systemic manifestations that can result in impaired functional capacity, worse dyspnea, reduced health-related quality of life, and increased mortality. The best-recognized manifestations include the presence of concomitant cardiovascular compromise, malnutrition (involving primarily the loss and dysfunction of skeletal muscles), osteoporosis, anemia, increased gastroesophageal reflux, and clinical depression and anxiety [18].

In a randomized clinical trial of glutamine ingestion prior to exercise, no beneficial effect on oxidative metabolism or performance during incremental and constant-load exercise in COPD patients was found [65]. In a randomized clinical trial of 40 patients receiving mechanical ventilation due to COPD exacerbation, blood levels of IL-6, IL-8, and tumor necrosis factor (TNF)-α were found to differ between patients receiving daily administration of a glutamine solution for 5 days *vs.* those receiving administration of a placebo for the same period [66]. In a study performed in patients with stable COPD, the effects of oral ingestion of glutamine and glutamate in metabolic disturbances were compared. Glutamine, but not glutamate, increased plasma concentrations of citrulline and arginine, substrates produced in the intestine and liver [67].

### 4.4. Glutamine in Cystic Fibrosis

Cystic fibrosis is a life-shortening autosomal recessive disease caused by mutations in the gene that encodes the CFTR, a protein with anion-channeling properties that participates in electrolyte homeostasis and fluid movement across mucosal surfaces. Undernutrition and failure to thrive are commonly found in children with CF. Contributing factors include anorexia associated with frequent infections, malabsorption due to exocrine pancreatic insufficiency, increased rates of energy expenditure or protein turnover, and the catabolic effects of corticosteroid treatment [68]. Undernutrition, in turn, adversely affects prognosis by impairing immune function and altering pulmonary function [17], as a consequence of a decrease in respiratory muscle strength [69].

In CF, the effects of glutamine alone or in association with growth hormone as protein anabolic agents were evaluated in two similar trials. In the first trial, children with CF and malabsorption secondary to exocrine pancreatic insufficiency received four weeks of oral glutamine (0.7 g/kg/day), subcutaneous human recombinant growth hormone (rhGH, 0.3 mg/kg/week), or a combination of both agents. Oral glutamine increased plasma glutamine concentrations, but had no measurable protein anabolic effects [70]. In the second study, children with CF who were either undernourished or had short stature received a similar treatment protocol. The results further suggested that oral glutamine supplementation may not affect body protein turnover in this population [71].

### 4.5. Paradoxical Effects of Glutamine in Lung Cancer

Lung cancer is the most common cancer and the leading cause of cancer-related mortality worldwide [72], and cigarette smoking is its most common etiological factor. Lung cancer is frequently seen in patients with COPD, and has been found to be the most frequent cause of death in patients with mild COPD [73].

Evidence supports a role of glutamine in reinforcing the pathological activity of cancer cell growth and maintenance of proliferative signaling pathways with increased autonomy relative to non-malignant cells [74]. As noted above, the dependency of lung cancer cells on glutamine for growth, survival, and cell progression has been well documented [34]. More recently, glutamine conversion into the tricarboxylic acid cycle intermediate alpha-ketoglutarate through glutaminase was shown to be essential for Kras-induced anchorage-independent growth in A549 cells [75]. Another study demonstrated that reductive carboxylation of glutamine is key for the metabolic reprogramming that enables cancer cells to survive and proliferative under hypoxia [76].

Studies have demonstrated the beneficial effects of glutamine administration as an agent with radio- and chemoprotective properties during cancer treatment without stimulating tumor growth [77]. Indeed, the toxic effects of chemotherapeutic agents and radiotherapy [77,78,79] further aggravate glutamine depletion in patients with cancer. Oral glutamine supplementation during concurrent chemoradiotherapy had a beneficial effect in preventing weight loss and reducing the severity and incidence of acute and late radiation-induced esophagitis [79]. Likewise, in lung cancer patients treated with thoracic radiotherapy, oral glutamine decreased the severity of acute radiotherapy-induced esophagitis [80].

### 4.6. The Lungs in Randomized Clinical Trials of Glutamine Supplementation in Critically Ill Patients

Critically ill patients have commonly a higher incidence of multiple organ failure, including lung compromise. However, the majority of studies investigating the effects of glutamine administration in critically ill patients have been performed to evaluate clinical outcomes such as 28-day mortality, in-hospital mortality, time to discharge from ICU/hospital, and infectious complications; few studies have specifically focused on the development of ventilator-associated pneumonia (VAP) and ARDS. In a French randomized controlled trial of ICU patients, glutamine-supplemented total parenteral nutrition reduced the rate of infectious complications, including the incidence of pneumonia [81]. Another randomized controlled trial aimed to determine whether glutamine-supplemented parenteral nutrition affects the nosocomial infection rate in surgical intensive care unit patients. A reduction in pneumonia rate and fewer days on mechanical ventilation were observed in patients who had undergone cardiac, vascular, and colonic surgeries [82]. Supplementation with the dipeptide alanyl-glutamine in critically ill trauma patients receiving standard enteral nutrition did not affect the incidence of VAP or the duration of mechanical ventilation [83]. In addition, no effect was observed on the expression and functionality of toll-like receptors or on the rate of respiratory infections in trauma patients admitted to an ICU who received parenteral glutamine supplementation [84]. The REDOX trial analyzed a combination of parenteral and enteral glutamine plus antioxidant supplementation. Patients were enrolled in the study if they were mechanically ventilated and had failure of two or more organs. Increase in-hospital stay, six-month mortality rate, and prolonged mechanical ventilation were observed with the use of glutamine [22]. The authors attributed these harmful effects to several factors including the high dose of glutamine prescribed and the enrollment of critically ill patients with multiorgan failure, several of whom were in shock, in comparison with previous studies. Indeed, a recent meta-analysis, including all trials mentioned above, suggested that glutamine supplementation given to a mixed population of critically ill patients does not affect primary outcome measures, such as hospital and ICU mortality. However, in subgroup analyses, infectious morbidity was reduced in patients receiving parenteral glutamine, for more than five days and with less severe disease. The authors speculated that glutamine does not have a protective effect on mortality in the most severe patients, since glutamine supplementation is not sufficient for recovery of organ dysfunction, which is the main determinant of death in these cases [12]. In contrast, the “GLND” trial, a prospective, randomized, controlled, double-blind, multicenter, phase III study that investigated the safety and clinical efficacy of glutamine dipeptide-supplemented parenteral nutrition showed that glutamine supplementation is safe, but did not improve clinical outcomes, as well as hospital infections, in surgical ICU patients after gastrointestinal, vascular, or cardiac surgery. Furthermore, there was no difference in median mechanical ventilation free days with glutamine supplementation [85].

The conflicting results of clinical trials that studied glutamine supplementation seem to be related to many factors, including: glutamine dose, beginning and duration of the treatment, route of glutamine administration, disease severity, plasma glutamine concentration at admission, and heterogeneity of the target population.

## 5. Conclusions

Respiratory diseases are usually accompanied by an enhanced inflammatory response and catabolic activity. The altered amino acid flux that occurs in these conditions may be reflected in changes in glutamine concentration and, consequently, in a systemic depletion of glutamine.

Exogenous glutamine administration might be beneficial in respiratory diseases, especially in ARDS, asthma, and during lung cancer treatment, thus representing a potential therapeutic tool in these conditions. Further experimental and large randomized clinical trials focusing on the development and progression of respiratory diseases are necessary to elucidate the effects and possible therapeutic role of glutamine in this setting.
